# Antibiotic Resistance Patterns and Molecular Characterization of Streptococcus suis Isolates from Swine and Humans in China

**DOI:** 10.1128/spectrum.00309-23

**Published:** 2023-05-08

**Authors:** Chang-Zhen Wang, Min-Ge Wang, Yue-Fei Chu, Ruan-Yang Sun, Jian-Guo Li, Xian-An Li, Jian Sun, Ya-Hong Liu, Yu-Feng Zhou, Xiao-Ping Liao

**Affiliations:** a Guangdong Laboratory for Lingnan Modern Agriculture, National Risk Assessment Laboratory for Antimicrobial Resistance of Animal Original Bacteria, College of Veterinary Medicine, South China Agricultural University, Guangzhou, China; b Guangdong Provincial Key Laboratory of Veterinary Pharmaceutics Development and Safety Evaluation, South China Agricultural University, Guangzhou, China; c Jiangsu Co-Innovation Center for the Prevention and Control of Important Animal Infectious Diseases and Zoonoses, Yangzhou University, Yangzhou, China; Yangzhou University

**Keywords:** *S. suis*, serotype, WGS, integrative and conjugative elements, ICEs, transferability

## Abstract

Streptococcus suis is a zoonotic pathogen that causes disease in humans after exposure to infected pigs or pig-derived food products. In this study, we examined the serotype distribution, antimicrobial resistance phenotypes and genotypes, integrative and conjugative elements (ICEs), and associated genomic environments of S. suis isolates from humans and pigs in China from 2008 to 2019. We identified isolates of 13 serotypes, predominated by serotype 2 (40/96; 41.7%), serotype 3 (10/96; 10.4%), and serotype 1 (6/96; 6.3%). Whole-genome sequencing analysis revealed that these isolates possessed 36 different sequence types (STs), and ST242 and ST117 were the most prevalent. Phylogenetic analysis revealed possible animal and human clonal transmission, while antimicrobial susceptibility testing indicated high-level resistance to macrolides, tetracyclines, and aminoglycosides. These isolates carried 24 antibiotic resistance genes (ARGs) that conferred resistance to 7 antibiotic classes. The antibiotic resistance genotypes were directly correlated with the observed phenotypes. We also identified ICEs in 10 isolates, which were present in 4 different genetic environments and possessed differing ARG combinations. We also predicted and confirmed by PCR analysis the existence of a translocatable unit (TU) in which the oxazolidinone resistance gene *optrA* was flanked by IS*1216E* elements. One-half (5/10) of the ICE-carrying strains could be mobilized by conjugation. A comparison of the parental recipient with an ICE-carrying transconjugant in a mouse *in vivo* thigh infection model indicated that the ICE strain could not be eliminated with tetracycline treatment. S. suis therefore poses a significant challenge to global public health and requires continuous monitoring, especially for the presence of ICEs and associated ARGs that can be transferred via conjugation.

**IMPORTANCE** S. suis is a serious zoonotic pathogen. In this study, we investigated the epidemiological and molecular characteristics of 96 S. suis isolates from 10 different provinces of China from 2008 to 2019. A subset of these isolates (10) carried ICEs that were able to be horizontally transferred among isolates of different S. suis serotypes. A mouse thigh infection model revealed that ICE-facilitated ARG transfer promoted resistance development. S. suis requires continuous monitoring, especially for the presence of ICEs and associated ARGs that can be transferred via conjugation.

## INTRODUCTION

Streptococcus suis is a serious zoonotic pathogen of swine that causes significant economic losses to the pig industry, posing a threat to human public health (1, 2). The clinical diseases of S. suis infection include meningitis, sepsis, endocarditis, arthritis, hearing loss, and skin lesions. Moreover, S. suis can cause opportunistic lower respiratory tract infections, i.e., pneumonia (3). Severe infections in humans result in high levels of mortality, while survival can be accompanied by lifelong sequelae such as deafness (4). The numbers of human cases of S. suis infection were low (1,600) and were concentrated primarily in Asia and Europe (4, 5). Two notable large-scale human infections occurred in China in 1998 and 2005, resulting in 14 and 35 deaths, respectively (6–8). Infection sources for humans have been pork and pork-derived food products, and these are considered high-risk factors (9). Since S. suis is a major swine pathogen, the monitoring of S. suis isolates on farms is necessary for animal as well as human health protection (10).

There are currently 29 serotypes of S. suis, and serotype 2 is the most common for both human and pig clinical isolates (4, 11). Clinical treatment relies on antibiotics since there is no available commercial vaccine (12, 13). The primary treatment modalities are penicillins alone or in combination with aminoglycosides, macrolides and lincosamides, fluoroquinolones, and tetracyclines (14). However, unreasonable antibiotic therapy has resulted in the increasing antibiotic resistance of S. suis (14–16). Thus, monitoring resistance in clinical isolates is strongly recommended.

Globally, the frequency of the acquisition of antibiotic resistance in S. suis has been lower than for common Gram-negative *Enterobacteriaceae* members, possibly due to the more limited horizontal transmission of antibiotic resistance genes (ARGs) in Gram-positive bacteria that rely on integrative and conjugative elements (ICEs) located in the chromosome (14). These elements are mobilized via circularization and can be horizontally self-transferred via conjugation (17). The primary functions of ICEs are in host bacterial evolution, and ICEs can transfer favorable fitness genes that enhance virulence, promote resistance to antibiotics and heavy metals, regulate biofilm formation, and encode toxin-antitoxin systems (18). S. suis isolates are also important antimicrobial resistance reservoirs that facilitate the spread of ARGs to major streptococcal pathogens, including Streptococcus pyogenes, Streptococcus pneumoniae, and Streptococcus agalactiae, and between isolates of different S. suis serotypes (19–21). Therefore, ICEs are primary mediators of ARG transfer, and a more complete understanding of their function is necessary to understand resistance development in S. suis.

## RESULTS

### Phylogenetic analysis of S. suis and typing of isolates.

**FIG 1:**
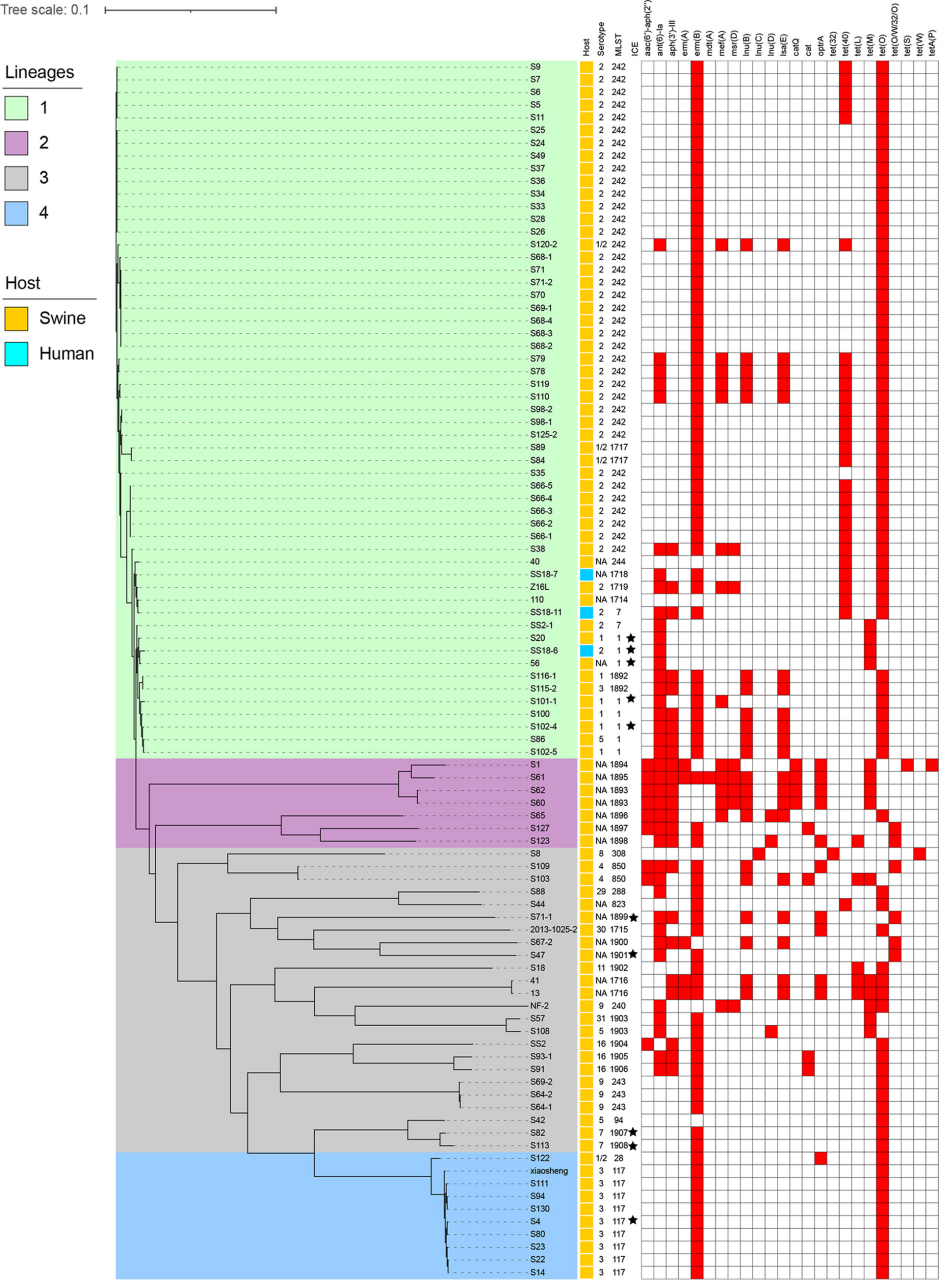
Analysis of 96 S. suis isolates identified in this study. The relationship between isolates is expressed as a maximum likelihood number, and yellow and green indicate different hosts. Red filling indicates the presence of ARGs, as indicated. MLST, multilocus sequence typing; NA, not applicable.

**FIG 2:**
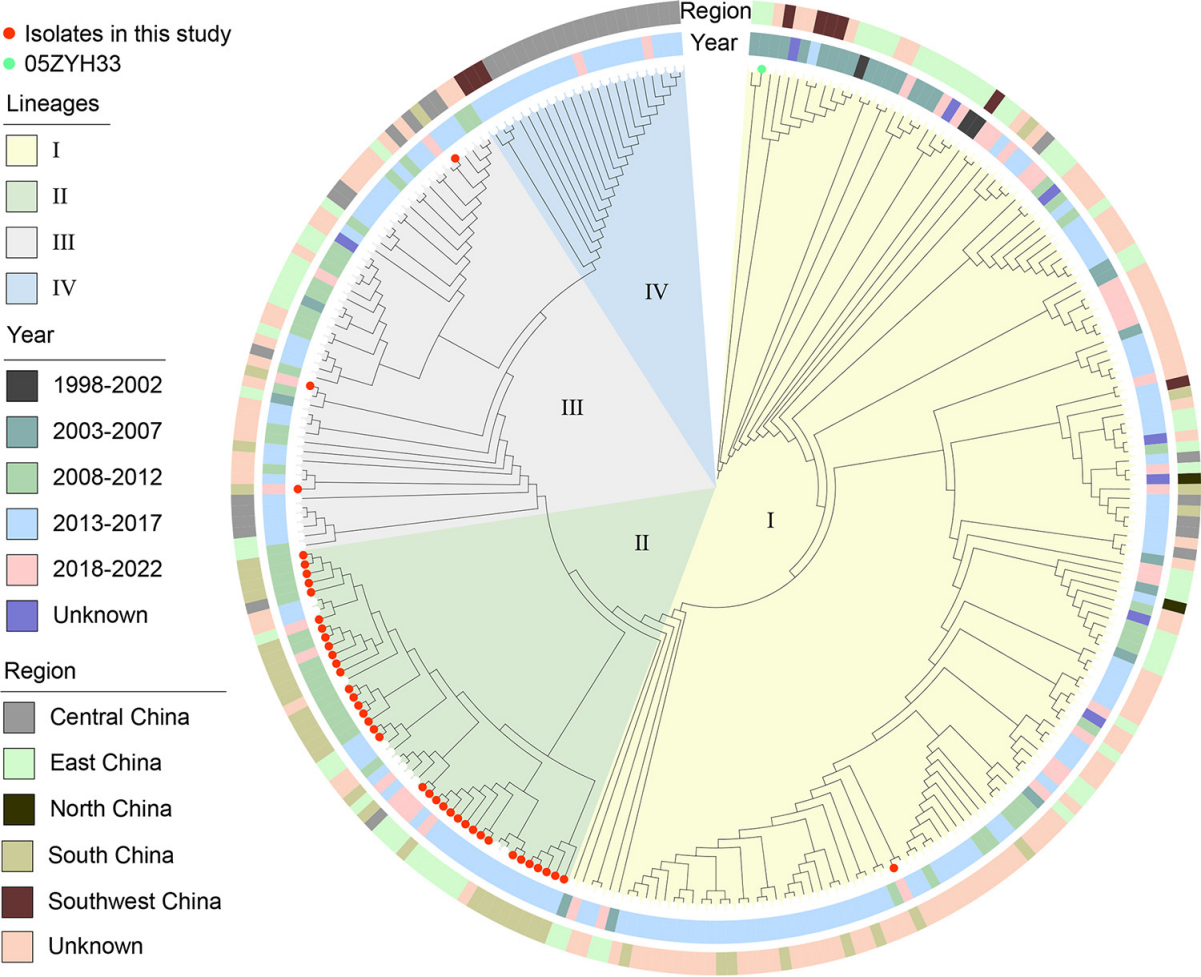
Phylogenetic structures of Chinese S. suis serotype 2 isolates from this study and the GenBank database. The maximum likelihood tree outlines the phylogenetic structures of 40 S. suis serotype 2 isolates unique to this study (highlighted by red points) combined with 232 Chinese S. suis serotype 2 isolates. The year of isolation and the location from which the isolates were obtained are indicated in the inner ring and the outer ring, respectively.

We examined all 96 of the S. suis isolates by whole-genome sequencing (WGS), and 79/96 (77.5%) included 13 known serotypes, including serotype 2 (40/96; 41.7%), serotype 3 (10/96; 10.4%), and serotype 1 (6/96; 6.3%). The remaining 10 serotypes involved fewer than 5% of the isolates. These 96 isolates contained a total of 36 sequence types (STs), and ST242 and ST117 predominated; interestingly, most ST242 isolates were of serotype 2, and all ST117 isolates were of serotype 3, while ST1714 to -1719 and ST1892 to -1908 were newly assigned STs (Fig. 1). A phylogenetic tree indicated the presence of 4 clades, and the major lineage I included 55 (57.3%) of the isolates. Further analysis revealed that lineage I included the 3 human isolates and possessed the broadest distributions by year and location, suggesting clonal spread. In particular, human isolate SS18-7 and porcine isolate 110 from Guangdong shared only 9 single nucleotide polymorphisms (SNPs), indicating a high probability of clonal transmission between pigs and humans (Fig. 1; see also Fig. S1 in the supplemental material). The phylogenetic tree demonstrated the molecular evolutionary relationship between the 40 S. suis serotype 2 (SS2) isolates in this study and the other 232 SS2 isolates in the NCBI database. The population structure analysis showed four distinct sequence lineages (named lineages I to IV), ranging in size from 22 to 152 isolates (Fig. 2). The majority of the 40 isolates in this study were assigned to lineage II. With the exception of 113 undetermined isolates, SS2 was the most abundant in eastern China but not in the northeastern and northwest regions. From 2013 to 2017, SS2 was the most abundant and was distributed across 4 lineages.

### Antimicrobial resistance profiles of S. suis isolates.

**FIG 3:**
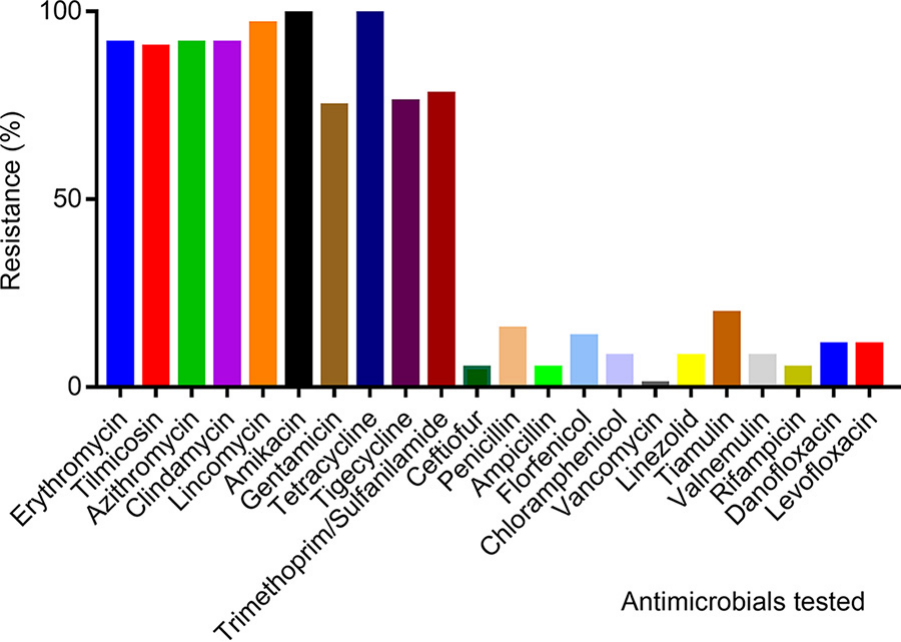
Summary of antibiotic resistance profiles of the 96 S. suis isolates used in this study.

The MICs were determined for 12 classes of antimicrobial agents, which included 22 antibiotics. All 96 isolates were resistant to amikacin and tetracycline, and high frequencies of resistance to erythromycin (91.7%), tilmicosin (90.6%), azithromycin (91.7%), clindamycin (91.7%), lincomycin (96.9%), trimethoprim-sulfanilamide (78.1%), tigecycline (76%), and gentamicin (75%) were observed. The rates of resistance to tiamulin, penicillin, florfenicol, danofloxacin, and levofloxacin were 19.8, 15.6, 13.5, 11.5, and 11.5%, respectively. Low frequencies of resistance to chloramphenicol (8.3%), linezolid (8.3%), valnemulin (8.3%), ceftiofur (5.2%), ampicillin (5.2%), rifampicin (5.2%), and vancomycin (1%) were observed (Fig. 3 and Fig. S2). These results corroborate the results of previous studies showing that S. suis was highly resistant to macrolides, tetracyclines, lincosamides, and aminoglycosides in China (15, 22, 23).

### Analysis of resistance profiles.

The group of 96 S. suis isolates possessed a total of 24 ARG types that conferred resistance to 7 classes of antibiotics, including macrolide-lincosamide-streptogramin B (MLS_B_), tetracycline, aminoglycosides, lincosamides, pleuromutilin-lincosamide-streptogramin A, chloramphenicol, and oxazolidinone. These resistance genotypes were consistent with the resistance phenotypes, and the MLS genes *erm*(B) and *tet*(O) were the most prevalent, at >80% (Fig. 1). These results suggest that this resistance profile might have been acquired by horizontal ARG transfer and could potentially mediate the development of multidrug resistance (MDR).

### ICE identification.

**FIG 4:**
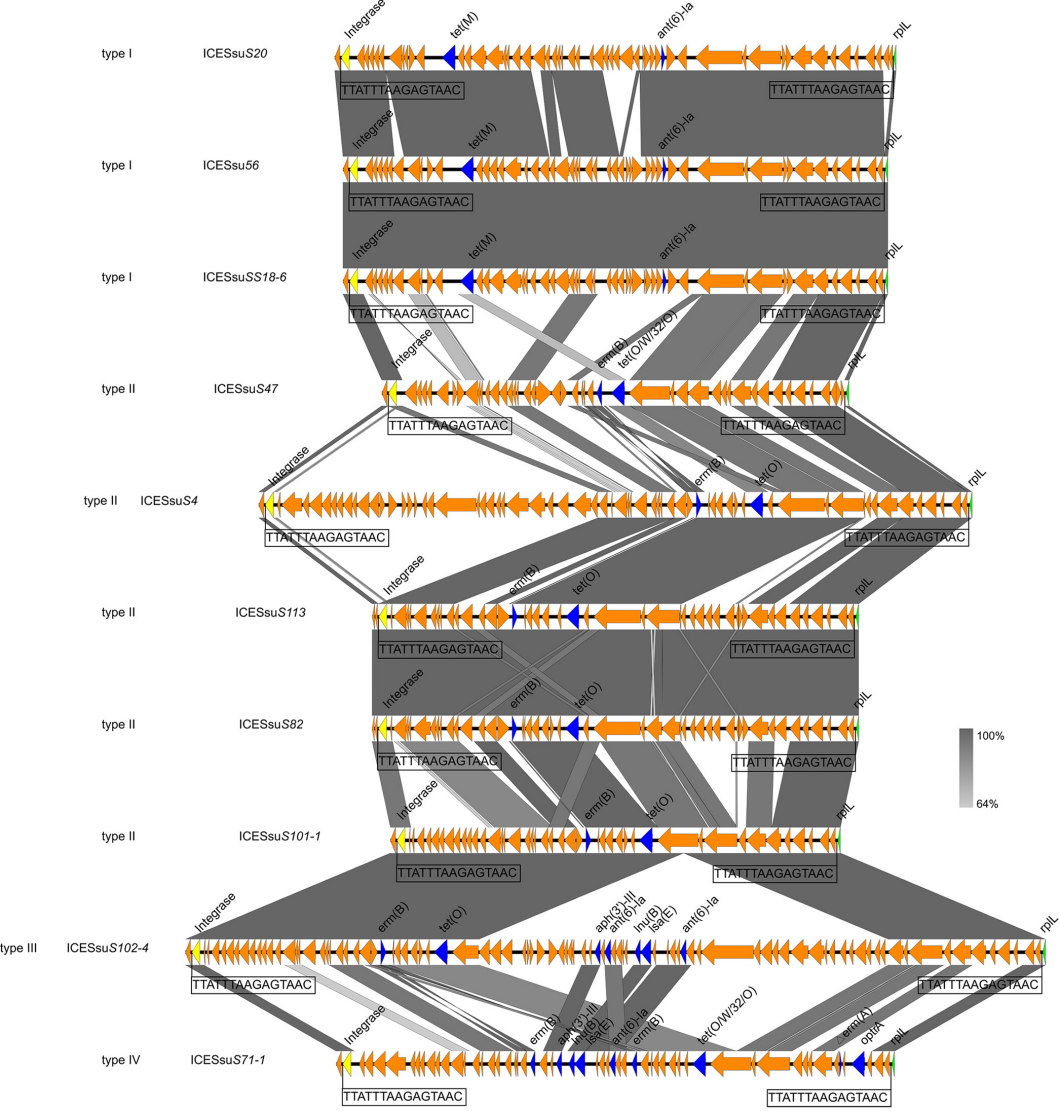
Identification of genetic environments (types I to IV) for ICEs in the S. suis isolates used in the study.

**FIG 5:**
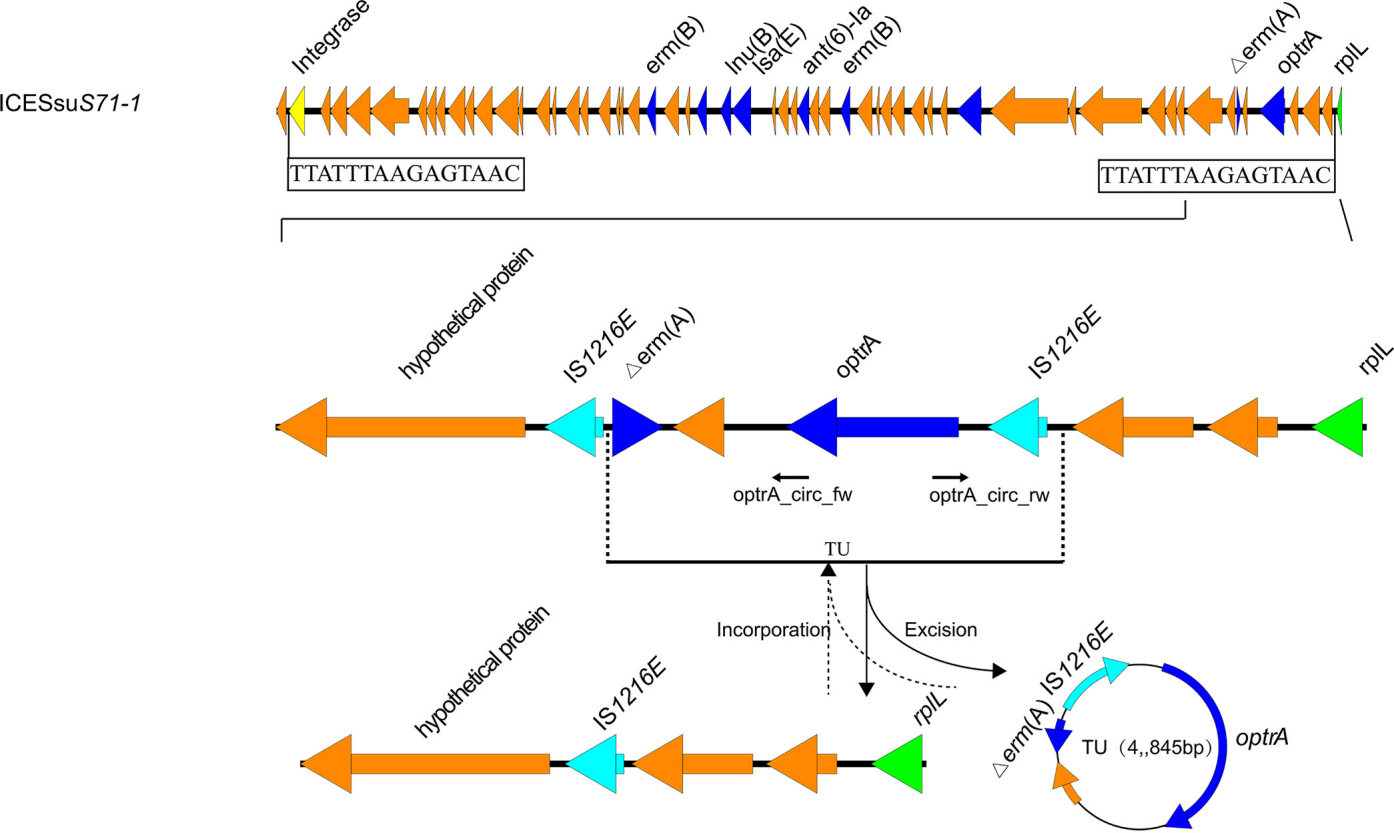
Location of the *optrA* gene in S. suis isolate S71-1. A TU carrying *optrA* was located on ICESsu*S71-1* in S. suis and identified using PCR analysis. Detection primers for the circularizable forms of the ICE (TU) are indicated by arrows. The Δ*erm* (A) indicates a truncated *erm* (A) gene.

ICEs and multidrug resistance have been previously reported for S. suis (20, 21, 24). In our group of 96 S. suis isolates, 10 were confirmed to be ICE carriers using WGS. These elements ranged in size from 64 to 124 kb, with GC contents of 35 to 38%. Each ICE was flanked by the 15-bp repeat 5′-TTATTTAAGAGTAAC-3′, indicative of *attL* and *attR* sites. The sites of integration for these 10 strains were located between the intergenic region extending from *hyd* (hydrolase, predicted) and *rplL* (50S large ribosomal protein subunit) (Table 1). A total of 4 genetic environments (types I to IV) were identified (Fig. 4). Type I (*n* = 3) carried *tet*(M) and *ant(6)-Ia*, and type II (*n* = 5) carried *erm*(B) and *tet*(O/W/32/O)-*tet*(M). Type III (*n* = 1) carried the gene cluster *aph(3′)-III*–*ant(6′)-Ia*–*lnu*(B)–*lsa*(E)–*ant(6)-Ia*. Type IV (*n* = 1) carried the truncated *erm* (A) gene [Δ*erm* (A)] and *optrA* genes, which were flanked by two copies of IS*1216E* in the same orientation, as well as some other genes (Fig. 5). These two copies of IS*1216E* could recombine and loop out a translocatable unit (TU) that consisted of one IS*1216E* copy along with the sequence located between the two IS*1216E* elements. A 4,845-bp TU was formed in ICESsu*S71-1* and contained one IS*1216E* copy and the ARGs Δ*erm*(A) and *optrA*, along with several other genes (Fig. 5).

**TABLE 1:** ICE-carrying S. suis isolates identified in this study

Strain	Serotype^*a*^	Multilocus ST	ICE size (bp)	GC content (%)	Insertions	Putative *att* sequence	Genotype
S20	1	ST1	80,799	37	*hyd* and *rplL*	TTATTTAAGAGTAAC	*ant(6)-Ia tet*(M)
S102-4	1	ST1	124,425	35	*hyd* and *rplL*	TTATTTAAGAGTAAC	*aph(3′)-III ant(6)-Ia erm*(B) *lnu*(B) *lsa*(E) *tet*(O)
S101-1	1	ST1	64,515	38	*hyd* and *rplL*	TTATTTAAGAGTAAC	*erm*(B) *tet*(O)
SS18-6	2	ST1	78,352	38	*hyd* and *rplL*	TTATTTAAGAGTAAC	*ant(6)-Ia tet*(M)
S4	3	ST117	103,002	38	*hyd* and *rplL*	TTATTTAAGAGTAAC	*erm*(B) *tet*(O)
S82	7	ST1907	69,887	38	*hyd* and *rplL*	TTATTTAAGAGTAAC	*erm*(B) *tet*(O)
S113	7	ST1908	69,806	38	*hyd* and *rplL*	TTATTTAAGAGTAAC	*erm*(B) *tet*(O)
56	NA	ST1	78,344	38	*hyd* and *rplL*	TTATTTAAGAGTAAC	*ant(6)-Ia tet*(M)
S71-1	NA	ST1899	80,342	37	*hyd* and *rplL*	TTATTTAAGAGTAAC	*aph(3′)-III ant(6)-Ia erm*(A) *erm*(B) *lnu*(B) *lsaE optrA tet*(O/W/32/O)
S47	NA	ST1901	66,984	37	*hyd* and *rplL*	TTATTTAAGAGTAAC	*tet*(O/W/32/O)

a NA, not applicable, indicating that the isolates were untypeable.

### Detection of the transferability of ICEs and difficult elimination of the recipient *in vitro* and *vivo*.

**FIG 6:**
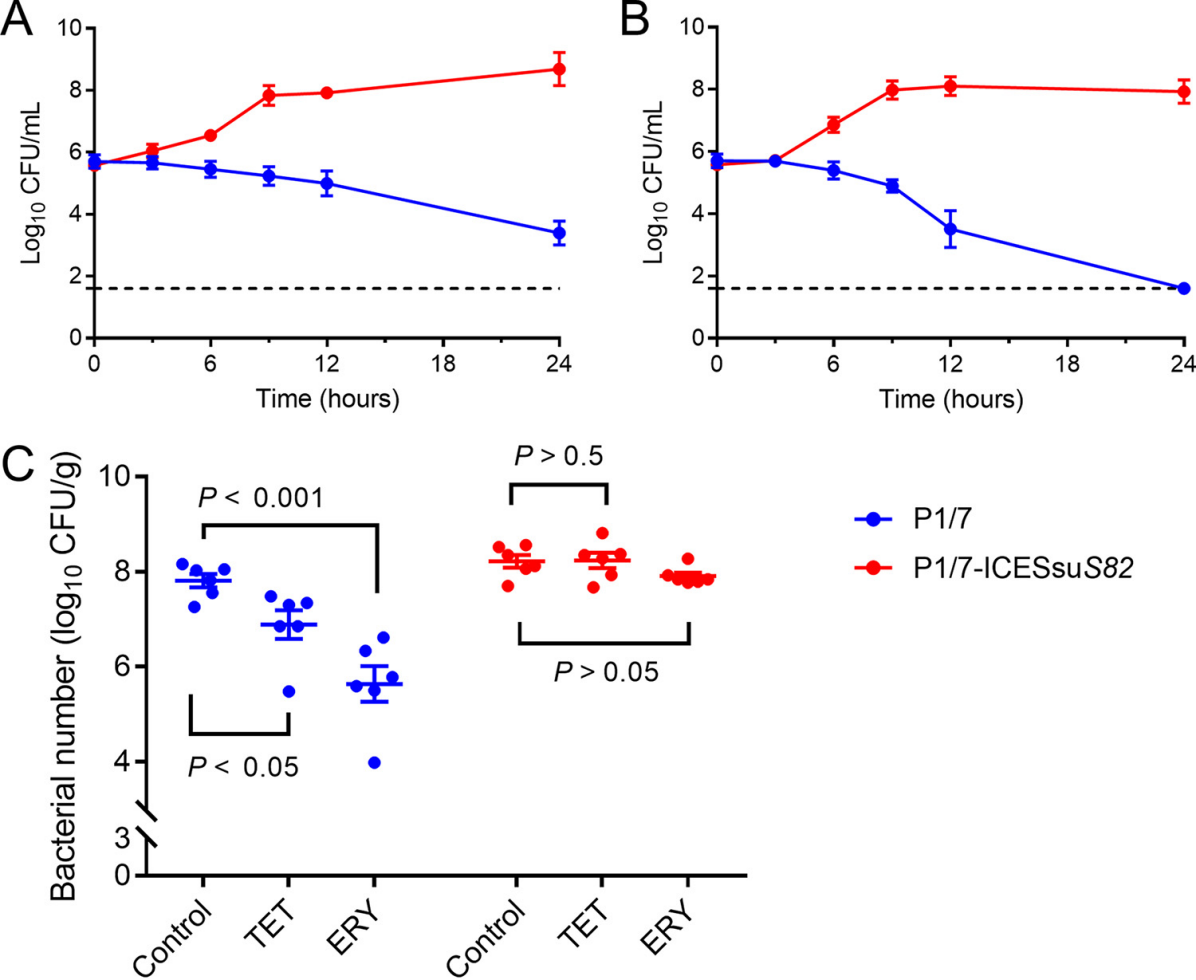
*In vitro* activities and *in vivo* therapeutic efficacies of tetracycline (TET) and erythromycin (ERY) against S. suis P1/7 and its transconjugant P1/7-ICESsu*S82*. (A and B) *In vitro* time-kill curves of tetracycline (A) and erythromycin (B) against S. suis strains. (C) *In vivo* therapeutic efficacy of tetracycline or erythromycin in the murine thigh infection model against S. suis strains.

Conjugation experiments were employed to assess the transferability of the ICEs in 10 S. suis isolates, and 5 were successfully transferred to the recipient S. suis P1/7 at frequencies ranging from 1.31 × 10^−8^ to 5.14 × 10^−8^. MIC testing indicated that the transconjugants possessed resistance phenotypes consistent with that of the donor (Table 2). Time-kill curves indicated that bacterial reductions of ~3 to 4 log CFU were observed for treatment with tetracycline or erythromycin against P1/7. However, the same treatment failed to inhibit P1/7-ICESsu*S82* (Fig. 6A and B). A transconjugant carrying *tet*(O) and *erm*(B) on ICESsu*S82* and unmodified recipient strain P1/7 were examined for pathogenicity using a mouse thigh infection model. Tetracycline or erythromycin treatment of mice infected with control strain P1/7 resulted in a significant 10-fold or 100-fold decrease in the CFU per gram of tissue, respectively (*P < *0.05). Conversely, tetracycline did not alter (*P > *0.05) the levels of the transconjugant P1/7-ICESsu*S82*, and the CFU were higher than those of the parental strain P1/7 (Fig. 6C). These results indicated that the ICE-carrying transconjugant possessed drug resistance that was difficult to eliminate *in vivo*.

**TABLE 2:** Characteristics of the strains included in the filter-mating conjugation experiments performed in this study

Strain	Serotype	Genotype	MIC (μg/mL)^*c*^			Transfer frequency
			RIF	TET	ERY	
P1/7^*a*^	2		>256	2	≤0.25	
S20	1	*ant(6)-Ia tet*(M)	≤0.0625	64	≤0.25	3.61 × 10^−8^
P1/7-ICESsu*S20*^*b*^	2		>256	64	≤0.25	
SS18-6	2	*ant(6)-Ia tet*(M)	≤0.0625	32	≤0.25	2.67 × 10^−8^
P1/7-ICESsu*SS18-6*^*b*^	2		>256	32	≤0.25	
S4	3	*erm*(B) *tet*(O)	≤0.0625	64	>256	3.72 × 10^−8^
P1/7-ICESsu*S4*^*b*^	2		>256	64	>256	
S82	7	*erm*(B) *tet*(O)	0.125	32	>256	5.14 × 10^−8^
P1/7-ICESsu*S82*^*b*^	2		>256	32	>256	
S113	7	*erm*(B) *tet*(O)	≤0.0625	32	>256	1.31 × 10^−8^
P1/7-ICESsu*S113*^*b*^	2		>256	32	>256	

a Recipient strain for transconjugant experiments.

b Transconjugant of the corresponding strain.

c RIF, rifampicin; TET, tetracycline; ERY, erythromycin.

## DISCUSSION

S. suis isolates are increasingly being recognized as the causes of preventable and emerging zoonotic infections in humans, with global distributions that are tracked using serotyping (25, 26). The S. suis serotypes identified in this study revealed substantial diversity, and for 83/96 isolates tested, we found 13 different capsular serotypes and 13.5% that were nontypeable. The nontypeable isolates may represent new serotypes of S. suis isolates, or they may be mutant variants of known serotypes with deletions and insertions in genes of the capsular polysaccharide locus (27). The Chinese isolates of highly pathogenic S. suis serotype 2 caused two large-scale human epidemics and massive pig deaths in China (28–30). Serotype 2 is also the most virulent serotype of S. suis isolates (31) and constitutes the bulk of published genomic sequences for S. suis (32). We also found that serotype 2 predominated in our isolates (40/96). We also identified ST1714 to -1719 and ST1892 to -1908 as newly assigned STs. However, the serotypes and STs did not exactly correspond, and a particular serotype could correspond to multiple STs and vice versa. However, all ST117 isolates were of serotype 3, and the majority of the ST242 isolates were of serotype 2. All isolates were classified into a 4-clade lineage, with lineage I covering the majority of the members across 7 years and 7 regions, suggesting clonal spread. In pigs, S. suis is transmitted primarily via aerosol, and airborne transmission between pigs has been clearly demonstrated (33). Human S. suis infections were almost always related to pig handling, pig slaughtering, or pork consumption (34) and might account for the presence of the 3 human isolates and the majority of the swine isolates in lineage I. Interestingly, a human isolate from Guangdong and a porcine isolate from Guangdong shared only 9 SNPs, and this was indicative of clonal transmission between animals and humans. Notably, Guangdong human isolate SS18-6, isolated in 2019, and 05ZYH33 (35), which caused the second large-scale outbreak of S. suis infection in China, were both part of lineage I. The harm of S. suis to humans should not be underestimated, and the spread of S. suis should not be ignored.

Antibiotics have long been used in the swine industry for disease treatment and prevention (14). However, antibiotics should be used with caution to reduce the selection of resistant S. suis isolates. MIC testing of our isolates indicated that the level of resistance of S. suis to commonly used antibiotics was relatively high. All isolates were resistant to at least one class of antibiotics, and high frequencies of resistance to amikacin, tetracycline, erythromycin, tilmicosin, azithromycin, clindamycin, and lincomycin were observed, consistent with the results of previous studies (15, 22). The resistance phenotypes of most of the isolates perfectly correlated with the presence of a corresponding acquired ARG. We found high rates of carriage of MLS_B_, tetracycline, and aminoglycoside resistance genes in our isolates. In veterinary medicine, macrolides and lincosamides can be used as feed and injection preparations for treating swine respiratory tract infections (14, 36). In addition, tetracycline, florfenicol, and penicillins are frequently applied separately or in combination for treating S. suis infections (37–39). Notably, we found linezolid and vancomycin resistance (LVR), and these are last-resort antimicrobial agents for the treatment of multidrug-resistant Gram-positive bacterial infections, which poses a severe threat to public health. LVR S. suis isolates have been previously described (40, 41). Therefore, close surveillance is urgently needed to monitor the antibiotic resistance of S. suis.

WGS analysis of our isolates further revealed that the macrolide resistance gene *erm* and the tetracycline resistance gene *tet* were carried at high frequencies, and this pattern was previously reported for S. suis (42, 43). Notably, resistance to the last-resort agent linezolid was carried by 11 of our isolates, indicated by the presence of *optrA*, which may have occurred under florfenicol selection on farms (40). Previous studies have established correlations between the presence of an ICE and multidrug resistance in S. suis (20, 24). We found 10 isolates that fit this pattern, and the type I and II resistance genes and the genetic environment were similar to those reported previously (20, 21, 44, 45); that is, the integration site was located in the intergenic region between *hyd* and *rplL*, similar to most reported ICE integration sites in S. suis (24, 46, 47). ICEs were inserted at the *rplL* locus, which is one of the common insertion hot spots for mobile genetic elements (MGEs) in S. suis, forming perfect 15-bp target site duplications at both termini (5′-TTATTTAAGAGTAAC-3′). In addition to the *rplL* locus, *rpsI* (S9 ribosomal protein), *lysS* (lysyl-tRNA), *rpmG* (L33 ribosomal protein), and *guaA* (GMP synthetase) have also been reported in Streptococcus spp. (46). We also identified additional resistance gene clusters for type III (ICESsu*S102-4*) and type IV (ICESsu*S71-1*). The *lsa*(E) gene has been found primarily as a part of a large MDR gene cluster often associated with *lnu*(B) (48). IS*26*-containing transposes have been shown to move via an excised circular element called a translocatable unit (TU) (49). The IS*26* mechanism also explains the properties of IS*1216*, which belongs to the same insertion sequence (IS) family and mobilizes resistance genes in Gram-positive bacteria (50, 51). Importantly, we found *optrA*, encoding lincosamide resistance, associated with the TU. This location will potentially aid in horizontal transfer among S. suis isolates and to other Gram-positive bacteria. We were able to successfully transfer the ICEs of isolates of 5 different serotypes to S. suis serotype 2 strain P1/7 by conjugation. Multiple attempts were unsuccessful for the remaining 5 isolates, and this failure was most likely associated with a lack of transfer elements in the donor strains, as previously reported (20, 45). Our *in vitro* and *in vivo* experiments further confirmed that ICEs carrying ARGs were difficult to eliminate with antibiotic treatment. As movable genetic elements, the ICEs in S. suis can be horizontally transferred to isolates of the same serotype and different serotypes of S. suis by conjugation. This can greatly accelerate ARG spread between different species and genera and could bring great difficulties and challenges for clinical treatment protocols.

### Conclusions.

We isolated 96 S. suis strains from humans and swine in China from 2008 to 2019, and serotype 2 predominated. WGS analysis verified that ST242 and ST117 were the most prevalent STs. Phylogenetic analysis of the core genomes of these isolates showed that S. suis most likely clonally spread between pigs and humans in China. WGS further revealed that the *erm*(B) and *tet*(O) genes were detected in more than 80% of the isolates. ICEs were detected in 10 S. suis isolates, which were divided into 4 types based on the genomic environment. ICEs can horizontally transfer ARGs between S. suis isolates of different serotypes. Our *in vivo* experiments further confirmed that an ICE strain possessing ARGs was difficult to eliminate clinically. Therefore, monitoring for ICEs as well as ARGs is necessary to ensure continued treatment efficacy for S. suis isolates.

## MATERIALS AND METHODS

### S. suis isolates and growth conditions.

We collected 96 S. suis isolates for the current study, including 3 human isolates, from the Guangdong Provincial Center for Disease Control and Prevention, and 93 isolates from swine farms in 10 provinces of China from 2008 to 2019 (see Table S1 in the supplemental material). Suspected S. suis isolates were grown, subcultured, and quantified using tryptic soy broth (TSB) and agar containing 5% newborn calf serum. All isolates were identified to the species level using matrix-assisted laser desorption ionization–time of flight (MALDI-TOF) mass spectrometry and 16S rRNA gene sequencing.

### Antimicrobial susceptibility testing.

Antimicrobial susceptibility testing was performed using the broth microdilution method according to 2022 European Committee on Antimicrobial Susceptibility Testing (EUCAST) (https://www.eucast.org/) guidelines. All S. suis isolates were tested for susceptibility to ceftiofur, penicillin, ampicillin, amikacin, gentamicin, erythromycin, tilmicosin, azithromycin, clindamycin, lincomycin, florfenicol, chloramphenicol, tetracycline, tigecycline, tiamulin, valnemulin, linezolid, vancomycin, rifampicin, danofloxacin, levofloxacin, and trimethoprim-sulfanilamide. The MIC results for ampicillin, rifampicin, and tigecycline were interpreted according to EUCAST breakpoints (52), and the MIC cutoffs for tilmicosin, lincomycin, amikacin, gentamicin, florfenicol, tiamulin, and valnemulin were reported based on a previous study (16), CLSI criteria were applied for the interpretation of the resistance profiles for the remaining antibiotics (53). Escherichia coli ATCC 25922 and Staphylococcus aureus ATCC 29213 were used as quality control strains.

### Whole-genome sequencing and analysis.

DNA was extracted from S. suis isolates using a commercial genomic DNA purification kit according to the manufacturer’s instructions (Tiangen, Beijing, China). Whole-genome sequencing (WGS) was performed with the Illumina HiSeq 2500 system (Novogene, Guangzhou, China) using the paired-end 2× 150-bp sequencing protocol. A total of 272 genomes of S. suis serotype 2 isolates that were originally isolated from China were used for phylogenetic and evolutionary analyses. The genomes of these isolates included 232 sequences retrieved from the NCBI database (https://www.ncbi.nlm.nih.gov/pathogens) (as of 10 March 2023) and 40 sequences generated during the present study. Assemblies from all isolates were mapped to the strain 05ZYH33 reference sequence (NCBI assembly accession number GCA_000014325.1) using Snippy v4.6.0 (https://github.com/tseemann/snippy). Recombinogenic SNPs were identified using Gubbins v2.4.1 (54). The phylogenetic tree was built with RAxML using the GTRGAMMA substitution model and 100 bootstraps and visualized using the iTOL online platform (55, 56). ARG homologs were identified using the ResFinder server (http://www.genomicepidemiology.org/) (57). Sequence types (STs) were identified using the PubMLST database (https://pubmlst.org/) (58). ICEs were predicted using ICEfinder (https://bioinfo-mml.sjtu.edu.cn/ICEberg2/index.php) (17), and complete ICE sequences on different contigs were determined using Sanger sequencing following PCR amplification (Table S2). Genomes were annotated using the Rapid Annotation of Microbial Genomes Using Subsystems Technology (RAST) annotation server (https://rast.nmpdr.org/rast.cgi). ICEs were identified by comparison with other MGEs from GenBank and were visualized using Easyfig 2.2.2 (59).

### Conjugation transfer experiment.

ARG transferability was assessed by conjugation using the rifampicin-resistant tetracycline-sensitive S. suis serotype 2 strain P1/7 as the recipient. Transconjugants were selected on tryptic soy agar (TSA) plates containing 10 μg/mL each of erythromycin and tetracycline and 30 μg/mL of rifampicin. The transconjugants were further examined for MIC phenotypes and by specific PCR amplification of ARGs and the unique serotype 2 gene sequences (Table S2) (60).

### Time-dependent killing.

P1/7 or P1/7-ICESsu*S82* bacterial cells at a final concentration of 1 × 10^6^ CFU/mL were treated with tetracycline (4 μg/mL) or erythromycin (4 μg/mL) for 0, 3, 6, 9, 12, and 24 h. At the indicated times, samples of each group were collected, diluted, and plated onto agar plates. After incubation at 37°C overnight, the CFU were calculated.

### Murine thigh infection model.

Six-week-old specific-pathogen-free ICR mice (23 to 29 g) were used for the *in vivo* studies (Guangdong Medical Lab Animal Center, Guangzhou, China). Mice were rendered neutropenic by the injection of cyclophosphamide intraperitoneally 4 days (150 mg/kg of body weight) and 1 day (100 mg/kg) prior to infection (61). Thigh infections were produced by the intramuscular injection of 0.10 mL of the inoculum (10^8^ CFU/mL of exponential-phase cells) into the thighs of isoflurane-anesthetized neutropenic mice. Two hours after thigh infection, the mice were randomized to receive (i) control treatment with the vehicle, (ii) tetracycline at 10 mg/kg by oral gavage twice a day, or (iii) erythromycin at 20 mg/kg by oral gavage twice a day. The dose of tetracycline was chosen according to a previous study with a standard oral dose in nursery pigs (62). The erythromycin dose was chosen to obtain pharmacokinetic values similar to those achieved with the clinical dose of 500 mg in humans (63–65). After 24 h of therapy, the thighs (six per group) were aseptically removed, homogenized, and processed for bacterial CFU determination.

### Ethics statement.

Animals were maintained according to national standards for laboratory animals in China (GB 14925-2010) (66). The Animal Research Committee (IACUC) of the South China Agricultural University (SCAU) approved these studies (approval number 2022C014).

## References

[1] Dong X, Chao Y, Zhou Y, Zhou R, Zhang W, Fischetti VA, Wang X, Feng Y, Li J. 2021. The global emergence of a novel *Streptococcus suis* clade associated with human infections. EMBO Mol Med 13:e13810. doi:10.15252/emmm.202013810. 34137500 PMC8261479

[2] Feng Y, Zhang H, Ma Y, Gao GF. 2010. Uncovering newly emerging variants of *Streptococcus suis*, an important zoonotic agent. Trends Microbiol 18:124–131. doi:10.1016/j.tim.2009.12.003. 20071175

[3] Gajdacs M, Nemeth A, Knausz M, Barrak I, Stajer A, Mestyan G, Melegh S, Nyul A, Toth A, Agoston Z, Urban E. 2020. *Streptococcus suis*: an underestimated emerging pathogen in Hungary? Microorganisms 8:1292. doi:10.3390/microorganisms8091292. 32847011 PMC7570012

[4] Huong VTL, Ha N, Huy NT, Horby P, Nghia HDT, Thiem VD, Zhu X, Hoa NT, Hien TT, Zamora J, Schultsz C, Wertheim HFL, Hirayama K. 2014. Epidemiology, clinical manifestations, and outcomes of *Streptococcus suis* infection in humans. Emerg Infect Dis 20:1105–1114. doi:10.3201/eid2007.131594. 24959701 PMC4073838

[5] Kerdsin A, Takeuchi D, Nuangmek A, Akeda Y, Gottschalk M, Oishi K. 2020. Genotypic comparison between *Streptococcus suis* isolated from pigs and humans in Thailand. Pathogens 9:50. doi:10.3390/pathogens9010050. 31936553 PMC7168618

[6] Lin L, Xu L, Lv W, Han L, Xiang Y, Fu L, Jin M, Zhou R, Chen H, Zhang A. 2019. An NLRP3 inflammasome-triggered cytokine storm contributes to streptococcal toxic shock-like syndrome (STSLS). PLoS Pathog 15:e1007795. doi:10.1371/journal.ppat.1007795. 31170267 PMC6553798

[7] Segura M. 2009. *Streptococcus suis*: an emerging human threat. J Infect Dis 199:4–6. doi:10.1086/594371. 19016626

[8] Ye C, Zheng H, Zhang J, Jing H, Wang L, Xiong Y, Wang W, Zhou Z, Sun Q, Luo X, Du H, Gottschalk M, Xu J. 2009. Clinical, experimental, and genomic differences between intermediately pathogenic, highly pathogenic, and epidemic *Streptococcus suis*. J Infect Dis 199:97–107. doi:10.1086/594370. 19016627

[9] Segura M, Calzas C, Grenier D, Gottschalk M. 2016. Initial steps of the pathogenesis of the infection caused by *Streptococcus suis*: fighting against nonspecific defenses. FEBS Lett 590:3772–3799. doi:10.1002/1873-3468.12364. 27539145

[10] Velikova N, Kavanagh K, Wells JM. 2016. Evaluation of *Galleria mellonella* larvae for studying the virulence of *Streptococcus suis*. BMC Microbiol 16:291. doi:10.1186/s12866-016-0905-2. 27978817 PMC5160000

[11] Okura M, Osaki M, Nomoto R, Arai S, Osawa R, Sekizaki T, Takamatsu D. 2016. Current taxonomical situation of *Streptococcus suis*. Pathogens 5:45. doi:10.3390/pathogens5030045. 27348006 PMC5039425

[12] Rieckmann K, Pendzialek S-M, Vahlenkamp T, Baums CG. 2020. A critical review speculating on the protective efficacies of autogenous *Streptococcus suis* bacterins as used in Europe. Porcine Health Manag 6:12. doi:10.1186/s40813-020-00150-6. 32391166 PMC7201539

[13] Segura M. 2015. *Streptococcus suis* vaccines: candidate antigens and progress. Expert Rev Vaccines 14:1587–1608. doi:10.1586/14760584.2015.1101349. 26468755

[14] Haenni M, Lupo A, Madec JY. 2018. Antimicrobial resistance in *Streptococcus* spp. Microbiol Spectr 6:ARBA-0008-2017. doi:10.1128/microbiolspec.ARBA-0008-2017. PMC11633561 29600772

[15] Zhang C, Zhang P, Wang Y, Fu L, Liu L, Xu D, Hou Y, Li Y, Fu M, Wang X, Wang S, Ding S, Shen Z. 2020. Capsular serotypes, antimicrobial susceptibility, and the presence of transferable oxazolidinone resistance genes in *Streptococcus suis* isolated from healthy pigs in China. Vet Microbiol 247:108750. doi:10.1016/j.vetmic.2020.108750. 32768204

[16] Wang X, Sun J, Bian C, Wang J, Liang Z, Shen Y, Yao H, Huang J, Wang L, Zheng H, Wu Z. 2021. The population structure, antimicrobial resistance, and pathogenicity of *Streptococcus suis cps31*. Vet Microbiol 259:109149. doi:10.1016/j.vetmic.2021.109149. 34147764

[17] Liu M, Li X, Xie Y, Bi D, Sun J, Li J, Tai C, Deng Z, Ou H-Y. 2019. ICEberg 2.0: an updated database of bacterial integrative and conjugative elements. Nucleic Acids Res 47:D660–D665. doi:10.1093/nar/gky1123. 30407568 PMC6323972

[18] Johnson CM, Grossman AD. 2015. Integrative and conjugative elements (ICEs): what they do and how they work. Annu Rev Genet 49:577–601. doi:10.1146/annurev-genet-112414-055018. 26473380 PMC5180612

[19] Chen L, Huang J, Huang X, He Y, Sun J, Dai X, Wang X, Shafiq M, Wang L. 2021. Horizontal transfer of different *erm*(B)-carrying mobile elements among *Streptococcus suis* strains with different serotypes. Front Microbiol 12:628740. doi:10.3389/fmicb.2021.628740. 33841355 PMC8032901

[20] Shang Y, Li D, Hao W, Schwarz S, Shan X, Liu B, Zhang S-M, Li X-S, Du X-D. 2019. A prophage and two ICESa2603-family integrative and conjugative elements (ICEs) carrying optrA in Streptococcus suis. J Antimicrob Chemother 74:2876–2879. doi:10.1093/jac/dkz309. 31314095

[21] Huang K, Song Y, Zhang Q, Zhang A, Jin M. 2016. Characterisation of a novel integrative and conjugative element ICE*SsD9* carrying *erm*(B) and *tet*(O) resistance determinants in *Streptococcus suis*, and the distribution of ICE*SsD9*-like elements in clinical isolates. J Glob Antimicrob Resist 7:13–18. doi:10.1016/j.jgar.2016.05.008. 27531000

[22] Yongkiettrakul S, Maneerat K, Arechanajan B, Malila Y, Srimanote P, Gottschalk M, Visessanguan W. 2019. Antimicrobial susceptibility of *Streptococcus suis* isolated from diseased pigs, asymptomatic pigs, and human patients in Thailand. BMC Vet Res 15:5. doi:10.1186/s12917-018-1732-5. 30606175 PMC6318959

[23] Wang M, Du P, Wang J, Lan R, Huang J, Luo M, Jiang Y, Zeng J, Quan Y, Shi Z, Zheng H. 2019. Genomic epidemiology of *Streptococcus suis* sequence type 7 sporadic infections in the Guangxi Zhuang Autonomous Region of China. Pathogens 8:187. doi:10.3390/pathogens8040187. 31614790 PMC6963630

[24] Zhu Y, Zhang Y, Ma J, Dong W, Zhong X, Pan Z, Yao H. 2019. ICESsuHN105, a novel multiple antibiotic resistant ICE in *Streptococcus suis* serotype 5 strain HN105. Front Microbiol 10:274. doi:10.3389/fmicb.2019.00274. 30863372 PMC6399138

[25] Qiu X, Bai X, Lan R, Zheng H, Xu J. 2016. Novel capsular polysaccharide loci and new diagnostic tools for high-throughput capsular gene typing in *Streptococcus suis*. Appl Environ Microbiol 82:7102–7112. doi:10.1128/AEM.02102-16. 27694240 PMC5118921

[26] Lun Z-R, Wang Q-P, Chen X-G, Li A-X, Zhu X-Q. 2007. *Streptococcus suis*: an emerging zoonotic pathogen. Lancet Infect Dis 7:201–209. doi:10.1016/S1473-3099(07)70001-4. 17317601

[27] Gurung M, Tamang MD, Moon DC, Kim S-R, Jeong J-H, Jang G-C, Jung S-C, Park Y-H, Lim S-K. 2015. Molecular basis of resistance to selected antimicrobial agents in the emerging zoonotic pathogen *Streptococcus suis*. J Clin Microbiol 53:2332–2336. doi:10.1128/JCM.00123-15. 25903569 PMC4473209

[28] Tang J, Wang C, Feng Y, Yang W, Song H, Chen Z, Yu H, Pan X, Zhou X, Wang H, Wu B, Wang H, Zhao H, Lin Y, Yue J, Wu Z, He X, Gao F, Khan AH, Wang J, Zhao G-P, Wang Y, Wang X, Chen Z, Gao GF. 2006. Streptococcal toxic shock syndrome caused by *Streptococcus suis* serotype 2. PLoS Med 3:e151. doi:10.1371/journal.pmed.0030151. 16584289 PMC1434494

[29] Yu H, Jing H, Chen Z, Zheng H, Zhu X, Wang H, Wang S, Liu L, Zu R, Luo L, Xiang N, Liu H, Liu X, Shu Y, Lee SS, Chuang SK, Wang Y, Xu J, Yang W, Streptococcus suis Study Groups. 2006. Human *Streptococcus suis* outbreak, Sichuan, China. Emerg Infect Dis 12:914–920. doi:10.3201/eid1206.051194. 16707046 PMC3373052

[30] Ye C, Zhu X, Jing H, Du H, Segura M, Zheng H, Kan B, Wang L, Bai X, Zhou Y, Cui Z, Zhang S, Jin D, Sun N, Luo X, Zhang J, Gong Z, Wang X, Wang L, Sun H, Li Z, Sun Q, Liu H, Dong B, Ke C, Yuan H, Wang H, Tian K, Wang Y, Gottschalk M, Xu J. 2006. *Streptococcus suis* sequence type 7 outbreak, Sichuan, China. Emerg Infect Dis 12:1203–1208. doi:10.3201/eid1708.060232. 16965698 PMC3291228

[31] Haas B, Grenier D. 2018. Understanding the virulence of *Streptococcus suis*: a veterinary, medical, and economic challenge. Med Mal Infect 48:159–166. doi:10.1016/j.medmal.2017.10.001. 29122409

[32] Shi Y, Zang N, Lou N, Xu Y, Sun J, Huang M, Zhang H, Lu H, Zhou C, Feng Y. 2022. Structure and mechanism for streptococcal fatty acid kinase (Fak) system dedicated to host fatty acid scavenging. Sci Adv 8:eabq3944. doi:10.1126/sciadv.abq3944. 36054360 PMC10848957

[33] Berthelot-Hérault F, Gottschalk M, Labbé A, Cariolet R, Kobisch M. 2001. Experimental airborne transmission of *Streptococcus suis* capsular type 2 in pigs. Vet Microbiol 82:69–80. doi:10.1016/s0378-1135(01)00376-5. 11423197

[34] Tarini NMA, Susilawathi NM, Sudewi AAR, Soejitno A, Fatmawati NND, Mayura IPB, Lestari AAW, Suputra G, Subrata IK, Astiti CISD, Besung INK, Mahardika GN. 2022. A large cluster of human infections of *Streptococcus suis* in Bali, Indonesia. One Health 14:100394. doi:10.1016/j.onehlt.2022.100394. 35686153 PMC9171533

[35] Feng Y, Zhang H, Wu Z, Wang S, Cao M, Hu D, Wang C. 2014. *Streptococcus suis* infection: an emerging/reemerging challenge of bacterial infectious diseases? Virulence 5:477–497. doi:10.4161/viru.28595. 24667807 PMC4063810

[36] Collignon PJ, Conly JM, Andremont A, McEwen SA, Aidara-Kane A, World Health Organization Advisory Group, Bogotá Meeting on Integrated Surveillance of Antimicrobial Resistance (WHO-AGISAR), Agerso Y, Andremont A, Collignon P, Conly J, Dang Ninh T, Donado-Godoy P, Fedorka-Cray P, Fernandez H, Galas M, Irwin R, Karp B, Matar G, McDermott P, McEwen S, Mitema E, Reid-Smith R, Scott HM, Singh R, DeWaal CS, Stelling J, Toleman M, Watanabe H, Woo G-J. 2016. World Health Organization ranking of antimicrobials according to their importance in human medicine: a critical step for developing risk management strategies to control antimicrobial resistance from food animal production. Clin Infect Dis 63:1087–1093. doi:10.1093/cid/ciw475. 27439526

[37] Chopra I, Roberts M. 2001. Tetracycline antibiotics: mode of action, applications, molecular biology, and epidemiology of bacterial resistance. Microbiol Mol Biol Rev 65:232–260. doi:10.1128/MMBR.65.2.232-260.2001. 11381101 PMC99026

[38] Wu T, Wang X, Dong Y, Xing C, Chen X, Li L, Dong C, Li Y. 2022. Effects of l-serine on macrolide resistance in *Streptococcus suis*. Microbiol Spectr 10:e00689-22. doi:10.1128/spectrum.00689-22. 35867475 PMC9430912

[39] Varela NP, Gadbois P, Thibault C, Gottschalk M, Dick P, Wilson J. 2013. Antimicrobial resistance and prudent drug use for *Streptococcus suis*. Anim Health Res Rev 14:68–77. doi:10.1017/S1466252313000029. 23683342

[40] Du F, Lv X, Duan D, Wang L, Huang J. 2019. Characterization of a linezolid- and vancomycin-resistant *Streptococcus suis* isolate that harbors *optrA* and *vanG* operons. Front Microbiol 10:2026. doi:10.3389/fmicb.2019.02026. 31551963 PMC6746840

[41] Ayobami O, Willrich N, Reuss A, Eckmanns T, Markwart R. 2020. The ongoing challenge of vancomycin-resistant *Enterococcus faecium* and *Enterococcus faecalis* in Europe: an epidemiological analysis of bloodstream infections. Emerg Microbes Infect 9:1180–1193. doi:10.1080/22221751.2020.1769500. 32498615 PMC7448851

[42] Chen L, Song Y, Wei Z, He H, Zhang A, Jin M. 2013. Antimicrobial susceptibility, tetracycline and erythromycin resistance genes, and multilocus sequence typing of *Streptococcus suis* isolates from diseased pigs in China. J Vet Med Sci 75:583–587. doi:10.1292/jvms.12-0279. 23292102

[43] Jiang F, Guo J, Cheng C, Gu B. 2020. Human infection caused by *Streptococcus suis* serotype 2 in China: report of two cases and epidemic distribution based on sequence type. BMC Infect Dis 20:223. doi:10.1186/s12879-020-4943-x. 32171281 PMC7071708

[44] Huang K, Zhang Q, Song Y, Zhang Z, Zhang A, Xiao J, Jin M. 2016. Characterization of spectinomycin resistance in *Streptococcus suis* leads to two novel insights into drug resistance formation and dissemination mechanism. Antimicrob Agents Chemother 60:6390–6392. doi:10.1128/AAC.01157-16. 27458226 PMC5038275

[45] Huang J, Ma J, Shang K, Hu X, Liang Y, Li D, Wu Z, Dai L, Chen L, Wang L. 2016. Evolution and diversity of the antimicrobial resistance associated mobilome in *Streptococcus suis*: a probable mobile genetic elements reservoir for other streptococci. Front Cell Infect Microbiol 6:118. doi:10.3389/fcimb.2016.00118. 27774436 PMC5053989

[46] Ambroset C, Coluzzi C, Guedon G, Devignes MD, Loux V, Lacroix T, Payot S, Leblond-Bourget N. 2015. New insights into the classification and integration specificity of *Streptococcus* integrative conjugative elements through extensive genome exploration. Front Microbiol 6:1483. doi:10.3389/fmicb.2015.01483. 26779141 PMC4701971

[47] Marini E, Palmieri C, Magi G, Facinelli B. 2015. Recombination between *Streptococcus suis* ICE*Ssu*32457 and *Streptococcus agalactiae* ICE*Sa*2603 yields a hybrid ICE transferable to *Streptococcus pyogenes*. Vet Microbiol 178:99–104. doi:10.1016/j.vetmic.2015.04.013. 25935120

[48] Yang M, Li X-S, Li D, Shang Y, Yu R, Schwarz S, Huang Z, Du X-D. 2020. Two novel *lsa*(E)-carrying mobile genetic elements in *Streptococcus suis*. J Antimicrob Chemother 75:2689–2691. doi:10.1093/jac/dkaa199. 32464646

[49] Harmer CJ, Hall RM. 2015. IS26-mediated precise excision of the IS26-aphA1a translocatable unit. mBio 6:e01866-15. doi:10.1128/mBio.01866-15. 26646012 PMC4676283

[50] Harmer CJ, Moran RA, Hall RM. 2014. Movement of IS*26*-associated antibiotic resistance genes occurs via a translocatable unit that includes a single IS*26* and preferentially inserts adjacent to another IS*26*. mBio 5:e01801-14. doi:10.1128/mBio.01801-14. 25293759 PMC4196232

[51] Zhu Y, Zhang W, Liu S, Schwarz S. 2021. Identification of an IS*431*-derived translocatable unit containing the *erm*(C) gene in *Staphylococcus aureus*. J Antimicrob Chemother 76:1102–1104. doi:10.1093/jac/dkaa555. 33428731

[52] European Committee on Antimicrobial Susceptibility Testing. 2020. Breakpoint tables for interpretation of MICs and zone diameters. Version 100.

[53] Clinical and Laboratory Standards Institute. 2015. Performance standards for antimicrobial susceptibility testing; 25th informational supplement. CLSI document M100-S25. Clinical and Laboratory Standards Institute, Wayne, PA.

[54] Croucher NJ, Page AJ, Connor TR, Delaney AJ, Keane JA, Bentley SD, Parkhill J, Harris SR. 2015. Rapid phylogenetic analysis of large samples of recombinant bacterial whole genome sequences using Gubbins. Nucleic Acids Res 43:e15. doi:10.1093/nar/gku1196. 25414349 PMC4330336

[55] Stamatakis A. 2006. RAxML-VI-HPC: maximum likelihood-based phylogenetic analyses with thousands of taxa and mixed models. Bioinformatics 22:2688–2690. doi:10.1093/bioinformatics/btl446. 16928733

[56] Letunic I, Bork P. 2019. Interactive Tree of Life (iTOL) v4: recent updates and new developments. Nucleic Acids Res 47:W256–W259. doi:10.1093/nar/gkz239. 30931475 PMC6602468

[57] Florensa AF, Kaas RS, Clausen PTLC, Aytan-Aktug D, Aarestrup FM. 2022. ResFinder—an open online resource for identification of antimicrobial resistance genes in next-generation sequencing data and prediction of phenotypes from genotypes. Microb Genom 8:e000748. doi:10.1099/mgen.0.000748. PMC8914360 35072601

[58] King SJ, Leigh JA, Heath PJ, Luque I, Tarradas C, Dowson CG, Whatmore AM. 2002. Development of a multilocus sequence typing scheme for the pig pathogen *Streptococcus suis*: identification of virulent clones and potential capsular serotype exchange. J Clin Microbiol 40:3671–3680. doi:10.1128/JCM.40.10.3671-3680.2002. 12354864 PMC130843

[59] Sullivan MJ, Petty NK, Beatson SA. 2011. Easyfig: a genome comparison visualizer. Bioinformatics 27:1009–1010. doi:10.1093/bioinformatics/btr039. 21278367 PMC3065679

[60] Liu Z, Zheng H, Gottschalk M, Bai X, Lan R, Ji S, Liu H, Xu J. 2013. Development of multiplex PCR assays for the identification of the 33 serotypes of *Streptococcus suis*. PLoS One 8:e72070. doi:10.1371/journal.pone.0072070. 23951285 PMC3739753

[61] Zhou Y-F, Tao M-T, He Y-Z, Sun J, Liu Y-H, Liao X-P. 2018. In vivo bioluminescent monitoring of therapeutic efficacy and pharmacodynamic target assessment of antofloxacin against Escherichia coli in a neutropenic murine thigh infection model. Antimicrob Agents Chemother 62:e01281-17. doi:10.1128/AAC.01281-17. PMC5740385 29038275

[62] Græsbøll K, Damborg P, Mellerup A, Herrero-Fresno A, Larsen I, Holm A, Nielsen JP, Christiansen LE, Angen Ø, Ahmed S, Folkesson A, Olsen JE. 2017. Effect of tetracycline dose and treatment mode on selection of resistant coliform bacteria in nursery pigs. Appl Environ Microbiol 83:e00538-17. doi:10.1128/AEM.00538-17. 28389548 PMC5452818

[63] Birkett DJ, Robson RA, Grgurinovich N, Tonkin A. 1990. Single oral dose pharmacokinetics of erythromycin and roxithromycin and the effects of chronic dosing. Ther Drug Monit 12:65–71. doi:10.1097/00007691-199001000-00012. 2305423

[64] Kovarik JM, Beyer D, Bizot MN, Jiang Q, Shenouda M, Schmouder RL. 2005. Effect of multiple-dose erythromycin on everolimus pharmacokinetics. Eur J Clin Pharmacol 61:35–38. doi:10.1007/s00228-004-0866-5. 15785960

[65] den Hollander JG, Knudsen JD, Mouton JW, Fuursted K, Frimodt-Møller N, Verbrugh HA, Espersen F. 1998. Comparison of pharmacodynamics of azithromycin and erythromycin in vitro and in vivo. Antimicrob Agents Chemother 42:377–382. doi:10.1128/AAC.42.2.377. 9527789 PMC105417

[66] Zhou YF, Tao MT, Feng Y, Yang RS, Liao XP, Liu YH, Sun J. 2017. Increased activity of colistin in combination with amikacin against Escherichia coli co-producing NDM-5 and MCR-1. J Antimicrob Chemother 72:1723–1730. 28333193 10.1093/jac/dkx038

