# Supplementary material for: Antibiotic Resistance Patterns and Molecular Characterization of Streptococcus suis Isolates from Swine and Humans in China
Source: Microbiol Spectr. 2023 May 8;11(3):e00309-23. doi: 10.1128/spectrum.00309-23 (PMC10269843; doi:10.1128/spectrum.00309-23)
Supplement: Supplemental file 1 — Supplemental material. Download spectrum.00309-23-s0001.pdf, PDF file, 0.4 MB [file spectrum.00309-23-s0001.pdf]

Supplementary Materials for

Antibiotic resistance patterns and molecular characterization of *Streptococcus suis* from swine and humans in China

Chang-Zhen Wang, Min-Ge Wang, Yue-Fei Chu, Ruan-Yang Sun, Jian-Guo Li, Xian-An Li, Jian Sun, Ya-Hong Liu, Yu-Feng Zhou, [#] and Xiao-Ping Liao, [#]

[#] To whom correspondence should be addressed: Yu-Feng Zhou, zyf@scau.edu.cn and Xiao-Ping Liao, xpliao@scau.edu.cn

The file includes:

Table S1. The information of *S. suis* isolates.

Table S2. Sequences of primers used for this study.

Figure S1. Heat map of SNP differences between human isolates and some swine isolates.

Figure S2. MIC distributions for the 96 *S. suis* isolates used in the current study.

Table S1. The information of *S. suis* isolates.

S. suis isolates	Source	Year	Locations	Lineages
110	Swine	2008	Guangdong, China	I
40	Swine	2008	Jiangxi, China	I
13	Swine	2009	Guangdong, China	III
S9	Swine	2011	Guangdong, China	I
S7	Swine	2011	Guangdong, China	I
S6	Swine	2011	Guangdong, China	I
S5	Swine	2011	Guangdong, China	I
S11	Swine	2011	Guangdong, China	I
S1	Swine	2011	Guangdong, China	II
S4	Swine	2011	Guangdong, China	IV
S38	Swine	2012	Guangdong, China	I
S49	Swine	2012	Hunan, China	I
S36	Swine	2012	Guangdong, China	I
S34	Swine	2012	Guangdong, China	I
S28	Swine	2012	Guangdong, China	I
S24	Swine	2012	Guangdong, China	I
SS2-1	Swine	2012	Jiangsu, China	I
S20	Swine	2012	Jiangsu, China	I
S44	Swine	2012	Guangdong, China	III
S47	Swine	2012	Fujian, China	III
S18	Swine	2012	Fujian, China	III
S57	Swine	2012	Anhui, China	III
S23	Swine	2012	Guangdong, China	IV
S22	Swine	2012	Jiangsu, China	IV
S14	Swine	2012	Guangdong, China	IV
S25	Swine	2012	Guangdong, China	I
S26	Swine	2012	Guangdong, China	I
S33	Swine	2012	Guangdong, China	I
S37	Swine	2012	Guangdong, China	I
S62	Swine	2013	Guangdong, China	II
S60	Swine	2013	Guangdong, China	II
S61	Swine	2013	Guangdong, China	II
2013-1025-2	Swine	2013	Shandong, China	III
S66-4	Swine	2015	Guangdong, China	I

Continue Table S1-1

S. suis isolates	Source	Year	Locations	Lineages
S66-2	Swine	2015	Guangdong, China	I
S66-1	Swine	2015	Guangdong, China	I
S71	Swine	2015	Anhui, China	I
S69-1	Swine	2015	Anhui, China	I
S68-2	Swine	2015	Anhui, China	I
S68-1	Swine	2015	Anhui, China	I
S65	Swine	2015	Guangdong, China	II
S67-2	Swine	2015	Shandong, China	III
S69-2	Swine	2015	Anhui, China	III
S64-1	Swine	2015	Zhejiang, China	III
xiaosheng	Swine	2015	Anhui, China	IV
S66-3	Swine	2015	Guangdong, China	I
S66-5	Swine	2015	Guangdong, China	I
S68-3	Swine	2015	Anhui, China	I
S68-4	Swine	2015	Anhui, China	I
S70	Swine	2015	Anhui, China	I
56	Swine	2016	Jiangsu, China	I
S8	Swine	2016	Anhui, China	III
NF-2	Swine	2016	Anhui, China	III
SS2	Swine	2016	Chongqing, China	III
Z16L	Swine	2017	Guangdong, China	I
S89	Swine	2017	Jiangsu, China	I
S84	Swine	2017	Hunan, China	I
S98-1	Swine	2017	Guangdong, China	I
S78	Swine	2017	Fujian, China	I
S86	Swine	2017	Jiangsu, China	I
S88	Swine	2017	Jiangsu, China	III
S93-1	Swine	2017	Chongqing, China	III
S91	Swine	2017	Jiangsu, China	III
S82	Swine	2017	Hunan, China	III
S80	Swine	2017	Fujian, China	IV
S94	Swine	2017	Guangdong, China	IV
S79	Swine	2017	Fujian, China	I
S98-2	Swine	2017	Guangdong, China	I
S119	Swine	2018	Anhui, China	I
S110	Swine	2018	Anhui, China	I

Continue Table S1-2

S. suis isolates	Source	Year	Locations	Lineages
S120-2	Swine	2018	Guangdong, China	I
S116-1	Swine	2018	Anhui, China	I
S115-2	Swine	2018	Anhui, China	I
S101-1	Swine	2018	Anhui, China	I
S100	Swine	2018	Anhui, China	I
S102-5	Swine	2018	Anhui, China	I
S102-4	Swine	2018	Anhui, China	I
S127	Swine	2018	Anhui, China	II
S123	Swine	2018	Shandong, China	II
S109	Swine	2018	Anhui, China	III
S103	Swine	2018	Anhui, China	III
S108	Swine	2018	Anhui, China	III
S113	Swine	2018	Guizhou, China	III
S122	Swine	2018	Shandong, China	IV
S111	Swine	2018	Anhui, China	IV
S125-2	Swine	2018	Shandong, China	I
SS18-11	Human	2019	Guangdong, China	I
SS18-7	Human	2019	Guangdong, China	I
SS18-6	Human	2019	Guangdong, China	I
S71-1	Swine	2019	Guangdong, China	III
41	Swine	2019	Guangdong, China	III
S64-2	Swine	2019	Zhejiang, China	III
S42	Swine	2019	Guangdong, China	III
S130	Swine	2019	Guangdong, China	IV
S35	Swine	2019	Guangdong, China	I
S71-2	Swine	2019	Guangdong, China	I

Table S2. Sequences of primers used for this study.

Primer designation	Sequence (5'-3')	Product size(bp)	Reference or source
erm (B)_fw	AAGTACTCAACCAAATAATA	651	This study
erm (B)_rv	ACAATACTTGCTCATAAGTA		
tet (M)_fw	TCTGCCGAAATTGTAATCAA	759	This study
tet (M)_rv	GTGCACTAATCACTTCCATT		
tet (O/W/32/O)_fw	GTGGATGGTATATTCTGCTT	1083	This study
tet (O/W/32/O)_rv	GTGGATGGTATATTCTGCTT		
*Cps 2*_fw	TTCGTATTAACTTACTTGGCGT	363	(1)
*Cps 2*_rv	TAAATCCCCATATGCCAAATCC		
ICESsu*S20*_fw	AATAGCCACGAGATGACACA	1648	This study
ICESsu*S20*_rv	GCGATAAGCGATTGATAGAA		
ICESsu*S4*-1_fw	ACTTGAAACTTGAATCAAAG	1743	This study
ICESsu*S4*-1_rv	TTCAATTAGTGACAGGTGTT		
ICESsu*S4*-2_fw	GATTAATCCACTGATGAGCC	386	This study
ICESsu*S4*-2_rv	AGGCTTTACGAGTTCAGAAG		
ICESsu*S4*-3_fw	GAATGGTGGTGTCAACTGAT	2569	This study
ICESsu*S4*-3_rv	AGACAGGGACAAGTCCACAA		
ICESsu*S4*-4_fw	TCTGGAACGGTATCTTCTAG	485	This study
ICESsu*S4*-4_rv	TTAGGCTAGGGTTGGAATCA		
ICESsu*S82*-1_fw	AACATGTGCCACTTTATCAT	2609	This study
ICESsu*S82*-1_rv	CATAAGATTAGTCACTGGTA		
ICESsu*S82*-2_fw	TGACTGCGATTAAACCTCCT	1410	This study
ICESsu*S82*-2_rv	ACGGTTGAGTCAATATGACA		
ICESsu*S82*-3_fw	ATTAATGAGGACACCATCCG	513	This study
ICESsu*S82*-3_rv	CCTTAACAGTTGATGCCTTA		
ICESsu*S82*-4_fw	AGTTGACCCTAAACTTTCAG	524	This study
ICESsu*S82*-4_rv	CTGATAATCAGGTCATCCAC		

Continue Table S2

Primer designation	Sequence (5'-3')	Product size(bp)	Reference or source
ICESsu*S82*-5_fw	CTTGCCATCCATAGGAGCCA	5212	This study
ICESsu*S82*-5_rv	GTGCTGGCGGATACTGAAGA		
ICESsu*S82*-6_fw	GCCATCAGATTTGAATTGGA	292	This study
ICESsu*S82*-6_rv	GATGAATTGGAACAAGTAGG		
ICESsu*S113*-1_fw	CGATTGCTTGGAATCAATGC	4609	This study
ICESsu*S113*-1_rv	CTGATAATCAGGTCATCCAC		
ICESsu*S113*-2_fw	GGGATTTCACTTGCAGATGT	3918	This study
ICESsu*S113*-2_rv	TCGAAAGAGTGATGAAATGC		
ICESsu*S113*-3_fw	TTCTGCACCTAGTCGATCAA	3785	This study
ICESsu*S113*-3_rv	ATACTCCGTCAGGTTTGATT		
ICESsu*S47*-1_fw	ATGTTCAGCTAAAGTGAAGG	1773	This study
ICESsu*S47*-1_rv	TGAGTGGTAACCAACTACAG		
ICESsu*S47*-2_fw	CCCTCCAAGACTAATATTCC	1284	This study
ICESsu*S47*-2_rv	GTTGTGGAGGTAATCATGCA		
*optrA*_circ_fw	TTGCGTTAGTACTAGCAATT	2955	This study
*optrA*_circ_rv	AATGGGAACAGTTGATGAGA		

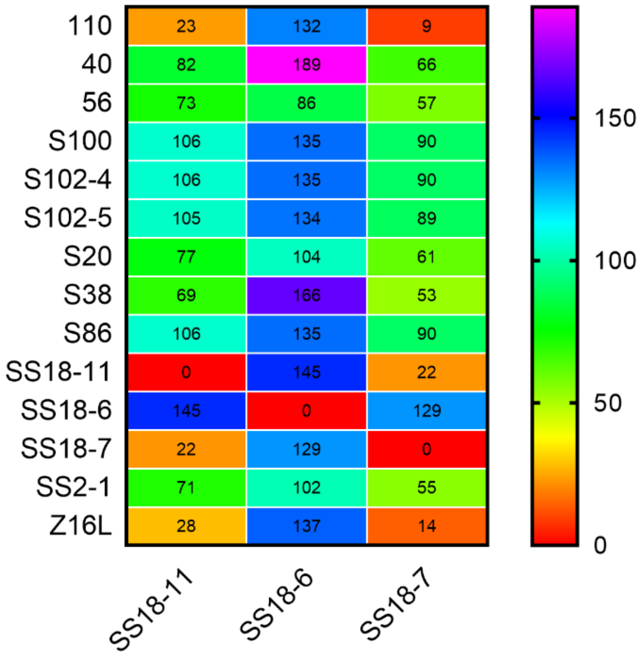

Figure S1. Heat map of SNP differences between human isolates and swine isolates in this study. The numbers represent SNPs shared between these two strain groups.

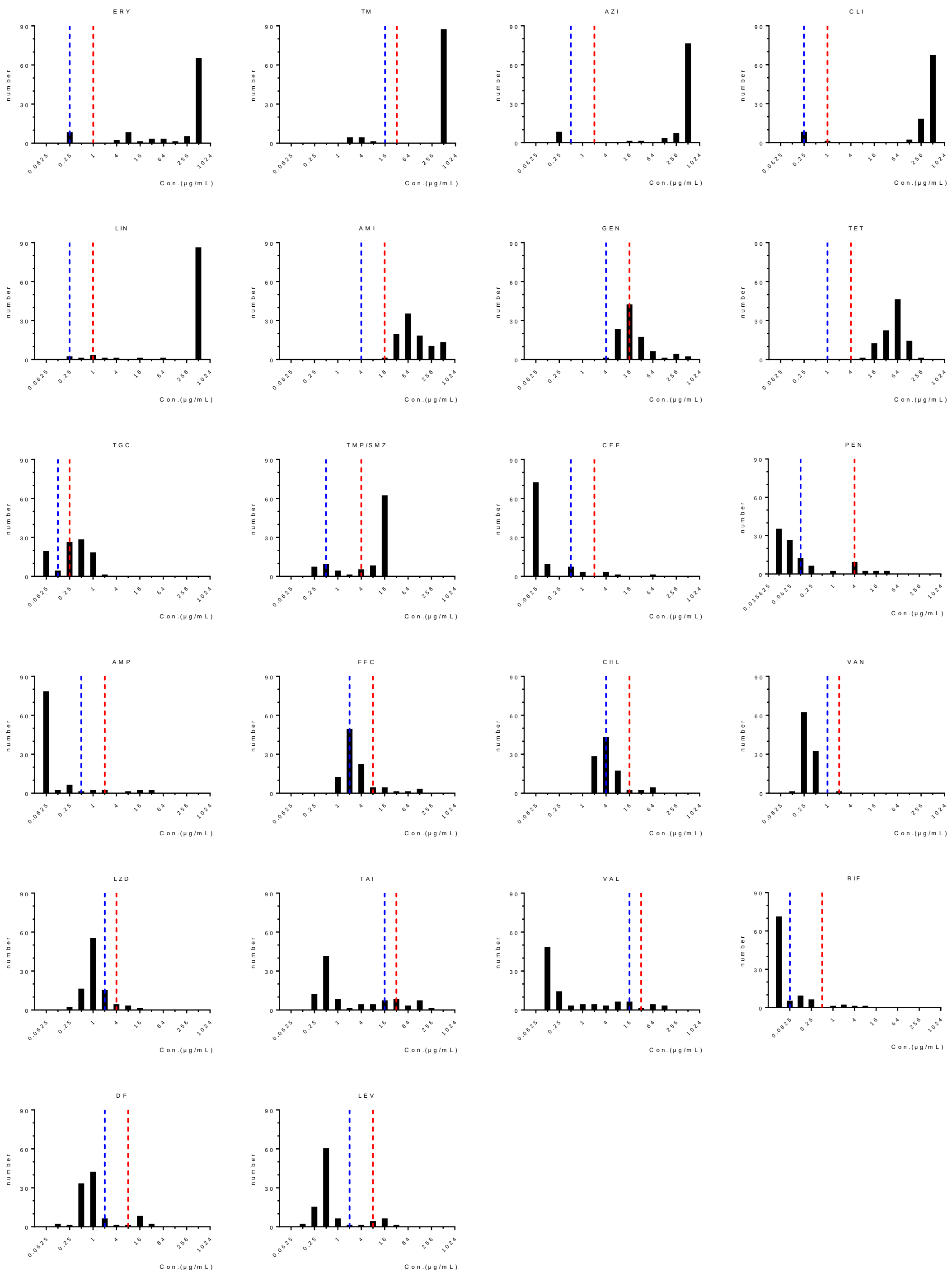

Figure S2. MIC distributions for the 96 *S. suis* isolates used in the current study. Blue and red lines indicate susceptible and resistant cutoffs, respectively. ERY, erythromycin; TM, tilmicosin; AZI, azithromycin; CLI, clindamycin; LIN, lincomycin; AMI, amikacin; GEN, gentamicin; TET, tetracycline; TGC, tigecycline; TMP/SMZ, trimethoprim/sulfanilamide; CEF, ceftiofur; PEN, penicillin; AMP, ampicillin; FFC, florfenicol; CHL, chloramphenicol; VAN, vancomycin; LZD, linezolid; TAI, tiamulin; VAL, valnemulin; RIF, rifampicin; DF, danofloxacin; LEV, levofloxacin. Except for

ampicillin, rifampin and tigecycline, which were interpreted using the EUCAST breakpoints (2) and tilmicosin, lincomycin, amikacin, gentamicin, florfenicol, tiamulin and valnemulin referred to a previous study (3), the CLSI criteria were applied for interpretation of the resistance profiles (4).

REFERENCES

1. Qiu X, Bai X, Lan R, Zheng H, Xu J. 2016. Novel Capsular Polysaccharide Loci and New Diagnostic Tools for High-Throughput Capsular Gene Typing in Streptococcus suis. Appl Environ Microbiol 82:7102-7112.
2. The European Committee on Antimicrobial Susceptibility Testing. 2020. Breakpoint tables for interpretation of MICs and zone diameters. version 100.
3. Wang X, Sun J, Bian C, Wang J, Liang Z, Shen Y, Yao H, Huang J, Wang L, Zheng H, Wu Z. 2021. The population structure, antimicrobial resistance, and pathogenicity of Streptococcus suis cps31. Vet Microbiol 259:109149.
4. Clinical and Laboratory Standards Institute. 2015. Performance standards for antimicrobial susceptibility testing; 25th informational supplement. CLSI document M100-S25 Clinical and Laboratory Standards Institute, Wayne, PA.
